# Interactive Impacts of Silver and Phosphorus on Autotrophic Biofilm Elemental and Biochemical Quality for a Macroinvertebrate Consumer

**DOI:** 10.3389/fmicb.2019.00732

**Published:** 2019-04-16

**Authors:** Clément Crenier, Kévin Sanchez-Thirion, Alexandre Bec, Vincent Felten, Jessica Ferriol, Aridane G. González, Joséphine Leflaive, Fanny Perrière, Loïc Ten-Hage, Michael Danger

**Affiliations:** ^1^Laboratoire Interdisciplinaire des Environnements Continentaux, Université de Lorraine – Centre National de la Recherche Scientifique, Metz, France; ^2^LTSER France, Zone Atelier du Bassin de la Moselle, Vandœuvre-lès-Nancy, France; ^3^Université Clermont Auvergne, CNRS UMR 6023, Laboratoire Microorganismes : Génome et Environnement, Clermont-Ferrand, France; ^4^Laboratoire Ecologie Fonctionnelle et Environnement, UMR 5245 CNRS-INP-UPS, Université de Toulouse, Toulouse, France; ^5^Instituto de Oceanografía y Cambio Global, Universidad de Las Palmas de Gran Canaria, Las Palmas de Gran Canaria, Spain

**Keywords:** ecological stoichiometry, essential fatty acids, trophic ecotoxicology, *Gammarus fossarum*, multiple stressors, benthic microalgae, freshwater biofilms

## Abstract

Autotrophic biofilms are complex and fundamental biological compartments of many aquatic ecosystems. In particular, these biofilms represent a major resource for many invertebrate consumers and the first ecological barrier against toxic metals. To date, very few studies have investigated the indirect effects of stressors on upper trophic levels through alterations of the quality of biofilms for their consumers. In a laboratory study, we investigated the single and combined effects of phosphorus (P) availability and silver, a re-emerging contaminant, on the elemental [carbon (C):nitrogen (N):P ratios] and biochemical (fatty acid profiles) compositions of a diatom-dominated biofilm initially collected in a shallow lake. We hypothesized that (1) P and silver, through the replacement of diatoms by more tolerant primary producer species, reduce the biochemical quality of biofilms for their consumers while (2) P enhances biofilm elemental quality and (3) silver contamination of biofilm has negative effects on consumers life history traits. The quality of biofilms for consumers was assessed for a common crustacean species, *Gammarus fossarum*, by measuring organisms’ survival and growth rates during a 42-days feeding experiment. Results mainly showed that species replacement induced by both stressors affected biofilm fatty acid compositions, and that P immobilization permitted to achieve low C:P biofilms, whatever the level of silver contamination. Gammarids growth and survival rates were not significantly impacted by the ingestion of silver-contaminated resource. On the contrary, we found a significant positive relationship between the biofilm P-content and gammarids growth. This study underlines the large indirect consequences stressors could play on the quality of microbial biomass for consumers, and, in turn, on the whole food web.

## Introduction

In ecosystems, living organisms most often face multiple stressors simultaneously. However, the interactive effects of these stressors, acting synergistically or antagonistically on species and ecosystems, remain hardly predictable. Particularly, in aquatic ecosystems, contaminants have often been described as co-occurring with eutrophication (Skei et al., 2000). However, the interactive effects of these stressors on ecosystem functioning remain poorly understood. By alleviating organisms’ nutrient limitation, increases in nutrient concentrations might potentially increase organisms’ tolerance to contaminants (Arce-Funck et al., 2016). In contrast, by altering water quality parameters, such as dissolved oxygen concentrations, one could expect changes in contaminant bioavailability (Skei et al., 2000) and/or intensification of the damages caused by the contaminants on already stressed organisms (Rattner and Heath, 2003). Finally, eutrophication-induced shifts in community composition could deeply change, either positively or adversely, the tolerance to contaminants of communities, mainly depending on individual species traits (Baird and Van den Brink, 2007).

In freshwater ecosystems, phosphorus (P) has long been recognized as one of the most important factor of eutrophication (Smith et al., 1999). Falkowski et al. (2000) have shown that human activities have increased the global fluxes of P by a fourfold factor when compared to natural fluxes, P exhibiting by far the most dysregulated biogeochemical cycle on earth. Since this chemical element is generally scarce in natural environments, any anthropogenic input is susceptible to deeply alter ecosystems functioning (Elser and Bennett, 2011). Due to these peculiarities, P-release impacts in aquatic ecosystems have received much attention in the past 60 years. Yet, these impacts have most often been considered without considering co-occurring stressors. Among the numerous contaminants entering aquatic ecosystems, toxic trace metals represent some of the most common persistent pollutants (Nriagu and Pacyna, 1988). Studying the interactive impacts of phosphorus load and toxic trace metal seems thus particularly important for understanding the impacts of contaminants in nature. Among the diversity of toxic metals that can be found in aquatic ecosystems, silver represents a reemerging contaminant that is commonly used as an antibacterial agent in partly soluble nanoparticles (Marambio-Jones and Hoek, 2010). Silver nanoparticles are currently among the most widely used nanoparticles (Project on Emerging Nanotechnology, 2012), and several studies predicted future increases in silver concentrations in surface waters due to its increased use (Geranio et al., 2009; Gottschalk et al., 2009). Concerning silver toxicity, silver is well known as a potent biocide, and is certainly among the most toxic to microorganisms (bacteria, fungi, and phytoplankton) and invertebrates (Ratte, 1999; Arce Funck et al., 2013a,b).

In numerous aquatic ecosystems, a large proportion of primary production is ensured by phototrophic biofilms. These biofilms are composed of attached communities generally dominated by microalgae, and containing different amounts of bacteria, fungi, and micro-eukaryotic species embedded in an organic polysaccharidic matrix (Wetzel, 1983). As a central basal resource, these biofilms play a fundamental role in aquatic food webs (Wetzel, 1983). These biofilms also have large impacts on aquatic biogeochemical cycles, ensuring the production, the decomposition, and the transfer of numerous organic molecules (Battin et al., 2003). Due to the ecological importance of biofilms, several studies have investigated the interactive impacts of diverse stressors on biofilm community structures and on some functional processes they ensure. For example, studies showed either antagonistic or synergistic effects of P concentration on the impacts of pesticides on biofilm community structure and/or primary production (Tlili et al., 2010; Murdock and Wetzel, 2012; Murdock et al., 2013). While being far less studied, some authors also showed that the deleterious impacts of metallic contaminants were reduced when biofilms were released from P limitation (Guasch et al., 2004; Serra et al., 2010). More recently, Leflaive et al. (2015) showed that high P concentrations alleviated the impacts of ionic silver on biofilm communities, but only for a cyanobacteria-dominated biofilm, impacts of silver on diatom-dominated biofilm being unaffected by P concentrations. Surprisingly, while multi-stressors impacts on biofilm community structure and functions have received much attention, and despite the major role of biofilms as basal resources in aquatic ecosystems, almost nothing is known on the cumulative impacts of multiple stressors on biofilm quality for its consumers. Yet, investigating this parameter might bring insightful results on multiple stressors indirect effects on aquatic food webs and, *in fine*, on ecosystem functioning.

In the literature, several parameters are generally evoked for measuring the potential quality of biofilms for their consumers. First, biofilm elemental content (often expressed as C:N:P ratios) has long been considered as an indicator of the quality of a resource for a consumer, this resource being of high quality when the imbalance between resource elemental content and consumers elemental requirements is minimal (Sterner and Elser, 2002). Since consumers have generally high N and P requirements, resources with the lowest C:P or C:N ratios are generally considered as resources of the highest quality, maximizing consumer life history traits such as growth rates or reproduction (Liess and Hillebrand, 2006; Danger et al., 2012, 2013). Second, the resource quality of a biofilm can also be evaluated by measuring its fatty acid content (a parameter classified as a biochemical quality parameter, see Crenier, 2017). Indeed, among the diversity of fatty acids found in nature, some of them are considered as essential for consumers, i.e., these fatty acids cannot be synthesized or at least not in sufficient amounts to fulfill the requirements of consumers (Arts et al., 2009). These essential fatty acids are generally composed of long chain polyunsaturated fatty acids (PUFAs), such as 20:5ω3 (EPA) and 22:6ω3 (DHA). The consumption of these compounds has been regularly reported as controlling consumers’ growth and/or reproduction (Masclaux et al., 2009; Crenier et al., 2017). Since fatty acid profiles are highly variable between algal groups, any change in biofilm algal communities can have drastic impacts on biofilm PUFA content (Muller-Navarra et al., 2004; Bec et al., 2010; Sanpera-Calbet et al., 2017). For example, the replacement of diatoms, rich in long-chain PUFAs, by green algae or cyanobacteria, depleted in such compounds, could have profound impacts on consumers’ life history traits and secondary production (Muller-Navarra et al., 2004). Finally, in the case of biofilms exposed to contaminants, the contaminant content of biofilm biomass could also be considered as a potential quality parameter since some contaminants can be highly toxic through trophic pathways (Luoma and Rainbow, 2008). Note that whatever the quality parameter investigated, effects of resources consumption on consumers life history traits must be systematically measured to evaluate the effective quality of resources, resource quality being not only dependent on resource composition but also on consumers requirements and physiology.

In the present study, we investigated the single and combined effects of P concentration and metallic contamination on biofilm quality for a model consumer, *Gammarus fossarum* (Crustacea, Amphipoda). This study is an independent part of the study published by Leflaive et al. (2015), dealing with the interactive impacts of P and ionic silver on biofilm (prokaryotic and microeukaryotic) communities. This study was carried out on a diatom-dominated biofilm initially collected in the field. First results showed that on this biofilm, both P increase and silver contamination led to significant reductions of diatoms proportion in algal communities, these algae being partly replaced by green algae (Leflaive et al., 2015). We thus hypothesize that P and silver lead to reductions in biofilm PUFA content, thus reducing biofilm biochemical quality for consumers (Hypothesis 1). In contrast, P increase in water lead to reductions of biofilm C:P ratios, thus potentially increasing resources elemental quality for consumers (Hypothesis 2). Finally, we hypothesize that silver has deleterious impacts on consumers’ growth and survival through the ingestion of toxic metal, leading to strong interactions between silver contamination and P concentration (Hypothesis 3).

## Materials and Methods

### Experimental Setup

To investigate the single and combined effects of Ag and P concentrations on biofilm quality, a diatom-dominated biofilm (diatoms representing 85–90% of algal biomass, Leflaive et al., 2015) was collected in the field. This biofilm was then exposed for 3 weeks in a full factorial design to a gradient of silver concentration at three distinct P concentrations. Impacts on microbial community structures were investigated independently from the present experiment, results being fully available in Leflaive et al. (2015). The present study specifically investigates the impacts of P and Ag stressors on biofilm fatty acids profiles, elemental composition, and silver concentration, giving an evaluation of biofilm potential quality for consumers. To measure the real/effective quality of biofilms, we fed a model consumer, *Gammarus fossarum*, with the distinct freeze dried biofilms, and followed organisms survival and growth throughout a 42-days experiment.

All details of the biofilm biomass production can be found in Leflaive et al. (2015). Briefly, the diatom-dominated biofilm was collected in a mesotrophic reservoir (Lake Saint-Ferréol, South-West France) using polyethylene plates (10 cm × 5 cm) placed vertically, 60-cm deep in the lake. Substrates were immerged during 3 weeks (May–June), then brought back to the laboratory in a cool box and placed two by two in beakers filled with 500 mL of modified COMBO medium (Kilham et al., 1998). The experiment was carried out in a controlled culture chamber (18°C, light intensity between 50 and 60 μE m^-2^ s^-1^, 16 h/8 h light/dark cycles). Twelve conditions were tested: three P levels (20, 100, or 500 μg/L) and four Ag concentrations (0, 5, 50, or 150 μg/L), each treatment being replicated three times (36 microcosms in total). Phosphorus concentrations were chosen as representative of what can be found along a gradient of P pollution in aquatic ecosystems (EEA, 2014). Concerning silver concentrations, total Ag concentrations have been shown to vary in natural waters between 0.03 and 500 ng L^-1^ (Luoma, 2008), but these concentrations are expected to increase in the future (Geranio et al., 2009; Gottschalk et al., 2009). To maintain levels of silver close to what could be found in contaminated rivers in our semi-continuous exposure conditions, we chose to expose biofilms to a 5 μg Ag L^-1^ concentration. The 50 and 100 μg Ag L^-1^ concentrations were retained as acute contamination levels, to evaluate the tolerance limits of biofilms to silver exposure. To limit too strong changes in our exposure conditions, media were entirely renewed each week. For that purpose, we emptied the beakers and re-filled them with newly prepared medium with the metal at the appropriate concentration. To limit silver adsorption on beakers surface, glass beakers were pre-saturated during 24 h with Ag solutions at concentrations similar to those of the diverse treatments.

### Sampling

Just after the introduction of biofilms in beakers (*t*_0_) and at the end of the experiment (after 3 weeks of biofilm growth, *t*_final_), 5 mL of medium from each beaker (*n* = 3 replicates per treatment, 36 samples in total per sampling date) were sampled, acidified with 15 μL of 70% HNO_3_ and stored at 4°C for later Ag quantification. At *t*_final_, biofilms present on polyethylene substrates were scrapped, then homogenized in 20 mL of incubation medium. Some subsamples were taken for measuring bacterial and micro-eukaryotic community structures. In particular, 1 mL was fixed (2% formaldehyde) and kept at 4°C for algal determination and counts (see Leflaive et al., 2015 for community structure results). The remaining volume of suspension was concentrated (centrifugation 7,000 × *g*, 10 min), supernatant was eliminated and the biofilm was stored at -20°C until being freeze-dried.

### Measurements of Ag in Biofilm and Water, and PO_4_^3-^ in Water

Silver concentrations in culture media was measured on *t*_0_ and *t*_final_ samples by atomic absorption spectrophotometry (graphite furnace Varian SpectrAA 300, detection limit = 0.1 μg Ag/L). The concentration of silver in the biofilm was measured after acidic digestion with 1 mL bidistilled HNO_3_ (15 N) and 1 mL H_2_O_2_ (30%, Sigma) for 24 h at 70°C. After full digestion, the samples were evaporated at 70°C and redissolved by 2% bidistilled HNO_3_ for analysis by ICP-MS (Agilent 7500 ce) with an uncertainty of 5% and a detection limit of 0.001 μg L^-1^. A reference standard solution SRM1646a (NIST, United States) was used to certify the accuracy and precision of the analytical procedure. The data quality was assessed by comparing the certified and the reference standard solution in terms of recovery (%), and by checking the precision of the ICP-MS analysis by the relative percentage differences (RPD) and the relative standard deviation (RSD) among the reference material replicates. The recovery for Ag was higher than 84.5% for all the samples. The precision of the instrument was within the 10% of RSD, thus was acceptable. All the materials used for silver sampling and measurements were cleaned with 1 N HCl for 24 h. Due to the cost of Ag analyses, measurements were done for each treatment on a pooled sample of biofilm corresponding to a mix of equal quantities of the three replicates (12 samples, i.e., 3 P-levels × 4 Ag concentrations). Initial PO_4_^3-^ concentrations were measured spectrophotometrically (ammonium molybdate method, AFNOR, 1990) in culture media used for microcosms filling.

### Biofilm Elemental Composition Measurements

Freeze-dried biofilm was first gently homogenized in a plastic centrifugation tube using a glass pestle. The C and N content of biofilm was measured on the 36 samples (3 P-levels × 4 Ag concentrations × 3 replicates) on ca. 1 mg samples using a CHN elementary analyzer (Carlo Erba NA2100, Thermo Quest CE International, Milan, Italy). Biofilm P content was quantified on the same number of samples after alkaline digestion with persulfate, and mineral P was then measured spectrophotometrically following the AFNOR (1990) procedure. Results are expressed as the mass percentage of the element in different resources, and C:N:P ratios correspond to molar ratios.

### Biofilm Fatty Acid Profiles Determination

Fatty acids analyses were performed on the 36 dried biofilm samples (3 P-levels × 4 Ag concentrations × 3 replicates). Analyses followed the procedure described in Crenier et al. (2017). Briefly, lipids were extracted twice with a chloroform/methanol solution according to the method proposed by Folch et al. (1957). Fatty acids were then converted into fatty acid methyl-esters (FAME) by acid catalyzed transesterification and analyzed on an Agilent Technologies^TM^ 6850 gas chromatograph. FAME were identified after a comparison of retention times with those obtained from Supelco^®^ and laboratory standards, and were quantified using internal standards (13:0).

### *Gammarus fossarum* Growth Measurements

#### Gammarids Sampling and Initial Sizing

Gammarids were collected in an unpolluted second-order forested stream (La Maix, Vosges Mountains Latitude N 48°29′02.1″, longitude E 007°04′008.5″). Organisms were immediately transported to the laboratory, then acclimatized at 12°C, in the dark and in aerated water for 15 days. The temperature of 12°C was chosen since preliminary observations showed that it permits to optimize gammarids growth while reducing risks of mortality increase due to water deoxygenation. Similarly, working in the dark permits to reduce the stress undergone by this light-avoiding species. During the acclimation period, animals were fed with alder [*Alnus glutinosa*, (L.) Gaertn.] leaf litter directly collected in La Maix stream. Two days before the beginning of the growth experiment, 204 gammarids of 4–5 mm in length were sorted and put in individual plastic cups containing 50 mL of La Maix stream water. Organisms were let emptying their guts for 48 h. During this period, all organisms were individually photographed, and initial sizes were measured with SigmaScan Image Analysis Version 5.000 (SPSS Inc., Chicago, IL, United States). The mean body length of gammarids used in the experiment was 4.52 ± 0.70 mm (mean ± SD), and size were similar between all treatments (ANOVA_2_: P effect: *F*_2,154_ = 0.3, *p* = 0.73; Ag effect: *F*_3,154_ = 0.3, *p* = 0.85; Ag × P effect: *F*_6,154_ = 1.7, *p* = 0.11).

#### Preparation of Biofilm Resources for *G. fossarum* Growth Experiment

Since measuring biofilm consumers growth take several weeks, and since biofilm quality can drastically change in a few weeks, we chose to feed the consumers with freeze-dried material, thus permitting to feed organisms with similar resource quality throughout the experiment (as proposed in Crenier et al., 2017). To maximize *G. fossarum* consumption of freeze-dried and homogenized biofilm, biofilm powders were embedded in low gelling temperature agarose (Sigma A9414), following the protocol proposed by Crenier et al. (2017). This agarose matrix permits to reconstitute a cohesive biofilm, and present the advantages of being nutrient-free. Agarose concentration used was 2%, and biofilm biomass introduced in each pellet was calculated to ensure that 50% of C came from the biofilm, the remaining coming from agarose. Agarose was dissolved in glass bottles with deionized water, heated in a microwave, and then placed in an agitated water bath at 38°C. After reaching this temperature, 1.6 ml of agarose solution was introduced in a 2 ml Eppendorf^®^ tube and mixed with biofilm powders. This mixture was then homogenized and poured in the holes of a Plexiglas^®^ mould (holes: 3 mm wide, 2 mm high). After a few minutes, the pellets were unmolded and kept at -20°C in Petri dishes until use.

#### *G. fossarum* Survival and Growth Experiment

Organisms were fed individually with one biofilm pellets. We verified that organisms were fed *ad libitum* and bioflim pellets were replaced every 2 days. The experiment lasted 42 days, and was carried out at 12°C, in the dark. Water was renewed every week, using aerated La Maix water. Survival was monitored daily. All survivors were photographed at the end of the experiment and the body length was measured as described above. The growth rates were expressed as length gain per mm of gammarid initial size per day (mm mm^-1^ d^-1^), as in Danger et al. (2013).

### Statistical Treatment of Data

All parameters (Ag concentration in water and biofilm, biofilm %C, %N, %P, C:N, C:P and N:P ratios, fatty acid contents, *Gammarus fossarum* growth rates) except survival curves were analyzed using two-way ANOVAs, considering P level and Ag concentrations as categorical predictors. Data were log transformed when necessary in order to meet the variance homoscedasticity condition for using ANOVAs. When ANOVA indicated significant differences between treatments, multiple comparisons were conducted using Tukey’s HSD test. Due to the elevated costs of analyses and the quantity of biological material required, Ag concentrations in biofilms were not replicated, rather measured on a pooled sample coming from the three replicates. The interactive effects of P and Ag were thus impossible to calculate, and only the main effects were analyzed. Survival were analyzed using Kaplan–Meier survival curves and log-rank tests for comparing curves. By accounting for censored data (i.e., when organisms were still alive at the end of the experiment), this approach enabled to investigate the effects of P and Ag during the whole experiment. Yet, since mortality remained null in several treatments of this experiment, it was statistically impossible to compare the 12 treatments. To make the analysis feasible, survival values were pooled by P levels, then by Ag levels. This permitted to compare the effects of P and Ag concentrations on gammarids survival, the analysis of the interactive effects of P and Ag being not possible. Survival curves analyses showing significant differences for both Ag and P treatments, the three curves obtained for P effect and the four curves obtained for Ag effects were compared pairwise. To take into account the multiple comparisons, *p*-values considered as significant followed a Bonferroni adjustment. Finally, impacts of resource quality parameters (biofilm C/P ratios, PUFA contents, and Ag concentrations) were analyzed using linear regressions. All statistical analyses were computed with STATISTICA (SAS institute). Statistical significance was inferred at *p* ≤ 0.05.

## Results

**FIGURE 1:**
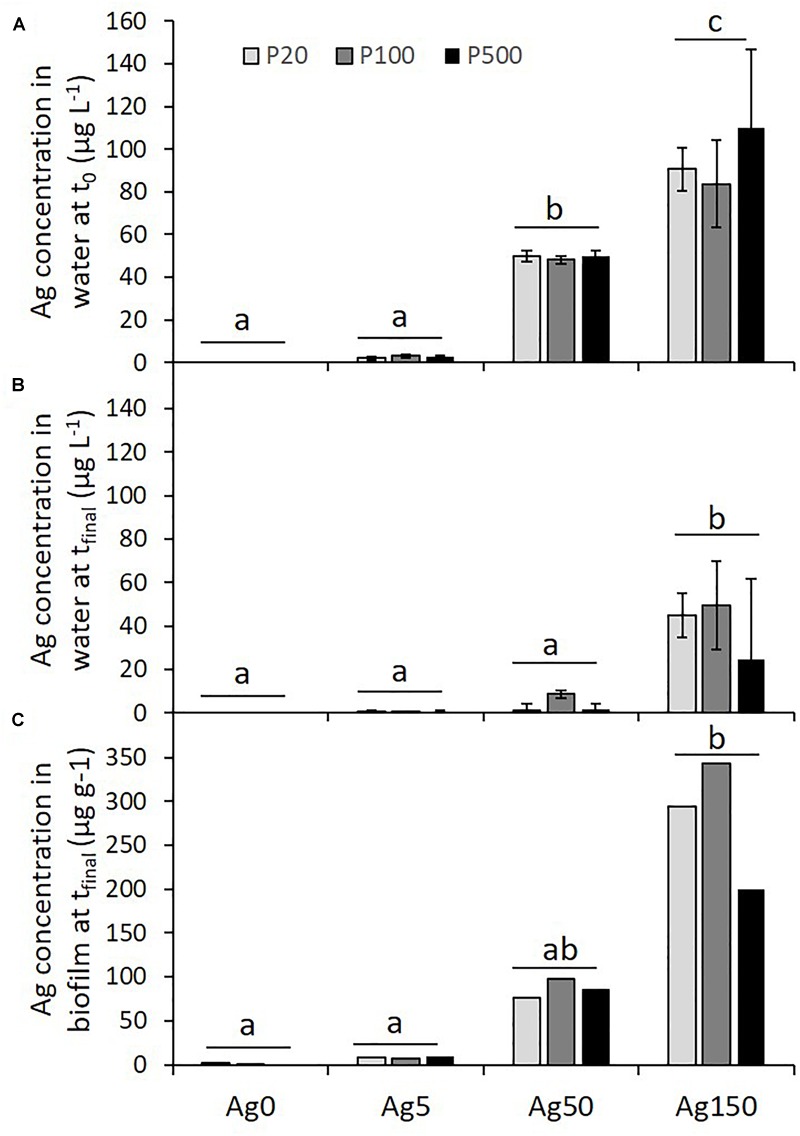
Concentrations of silver in water **(A,B)** and in biofilm **(C)** in the diverse Ag and P treatments. Different letters indicate significant differences after *post hoc* Tukey tests.

The measured Ag concentrations after microcosms filling (*t*_0_) were generally slightly lower than the nominal concentrations, these concentrations reaching 0.00 ± 0.00, 2.63 ± 0.29, 49.42 ± 0.75, and 94.67 ± 8.22 μg Ag L^-1^ in the 0, 5, 50, and 150 μg Ag L^-1^ treatments, respectively (Figure 1A). The Ag concentration strongly decreased after 1 week in the presence of biofilm (*t*_final_) (Figure 1B). The Ag concentration achieved 0.00 ± 0.00, 0.31 ± 0.08, 3.91 ± 1.99, and 39.58 ± 6.31 μg Ag L^-1^ with respect to the initial expected levels (0, 5, 50, and 150 μg Ag L^-1^). At both *t*_0_ and *t*_final_, Ag concentrations were totally independent of the P concentration, as revealed by the absence of significant interaction between Ag and P concentrations (Figure 1A,B and Table 1). Ag measurements in biofilm showed traces of Ag in control biofilm (0.8 ± 1.11 μg g^-1^), while Ag concentrations reached 8.24 ± 1.19, 86.41 ± 10.77, and 279.13 ± 72.85 μg g^-1^, in the 5, 50, and 150 μg Ag L^-1^ treatments, respectively (Figure 1C and Table 1).

**Table 1:** Results of the two-way ANOVAs conducted on the main endpoints for investigating the effects of P concentrations, Ag levels, and their interactive effects.

Parameter	Two-way ANOVA									
	P concentration			Ag concentration			P × Ag			d.f. error
	d.f.	*F*	*p*-Value	d.f.	F	*p*-Value	d.f.	*F*	*p*-Value	
[Ag] in water at *t*_0_	2	0.9	0.41	3	113.7	**<0.001**	6	0.8	0.55	24
[Ag] in water at *t*_final_	2	2.3	0.12	3	39.8	**<0.001**	6	1.5	0.21	24
[Ag] in biofilm at *t*_final_	2	1.1	0.39	3	37.9	**<0.001**	na	na	na	6
**Elemental quality**										
%C	2	0.3	0.76	3	0.6	0.64	6	1.4	0.26	24
%N	2	0.8	0.44	3	2.2	0.10	6	0.7	0.61	24
%P	2	129.8	**<0.001**	3	1.0	0.39	6	0.8	0.59	24
*C:N ratio*	2	5.1	**0.01**	3	3.5	**0.03**	6	1.6	0.19	24
*C:P ratio*	2	369.1	**<0.001**	3	3.2	**0.04**	6	1.3	0.29	24
*N:P ratio*	2	374.8	**<0.001**	3	0.8	0.52	6	1.8	0.14	24
**Fatty acid profiles**										
*SAFA*	2	0.2	0.81	3	3.8	**0.02**	6	1.7	0.17	23
*MUFA*	2	14.5	**<0.001**	3	2.7	0.06	6	2.1	0.08	23
*PUFA*	2	4.9	**0.01**	3	1.8	0.16	6	1.2	0.32	23
*18:3ω3*	2	1.8	0.19	3	6.4	0.003	6	1.0	0.44	23
*20:5ω3*	2	2.9	0.06	3	1.9	0.15	6	1.8	0.12	23
*Sum fatty acids*	2	0.1	0.98	3	4.1	**0.02**	6	1.9	0.12	23
*G. fossarum* growth rates	2	2.9	0.05	3	0.6	0.60	6	0.7	0.60	154

*The columns d.f. and *F* correspond to the number of degree of freedom and the F-statistic in the two-way ANOVA test, respectively. Significant differences are indicated in bold.*

**FIGURE 2:**
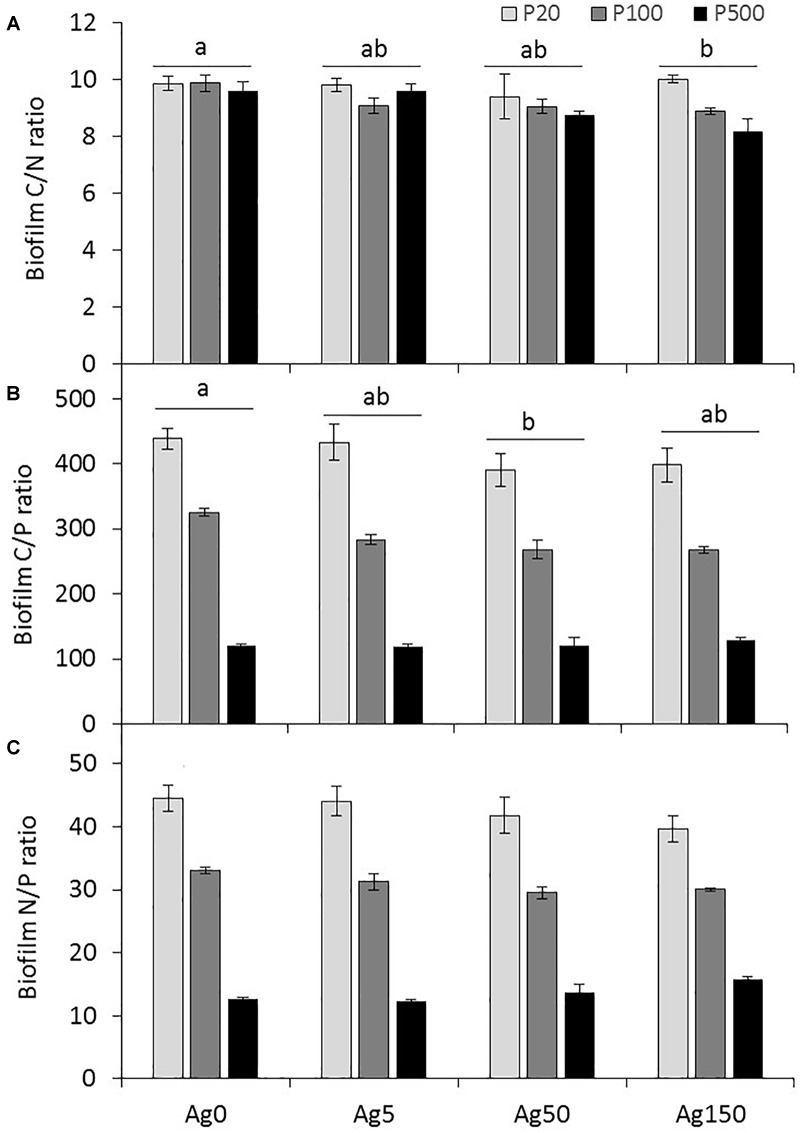
**(A–C)** Elemental ratios (molar ratios) in biofilm biomass for the different P and Ag treatments. Significant differences due to Ag levels after *post hoc* Tukey tests are shown by different letters. No interactive effects were evidenced.

Results of biofilm elemental composition measurements showed that biofilm %C and %N remained unchanged whatever the P and Ag concentrations tested (Figure 2 and Table 1). In contrast, biofilm %P was strongly altered by the P concentration in water, but remained unchanged along the Ag gradient (no interactive effect). All elemental ratios were modified by P concentration in the culture medium, biofilm C:P, C:N, and N:P ratios being significantly reduced under the highest P-concentrations. In contrast, only biofilm C:N and C:P ratios were significantly altered by the Ag concentration, these ratios slightly decreasing along the Ag concentration gradient (Figure 2), and no interactive effect was revealed (Table 1).

**FIGURE 3:**
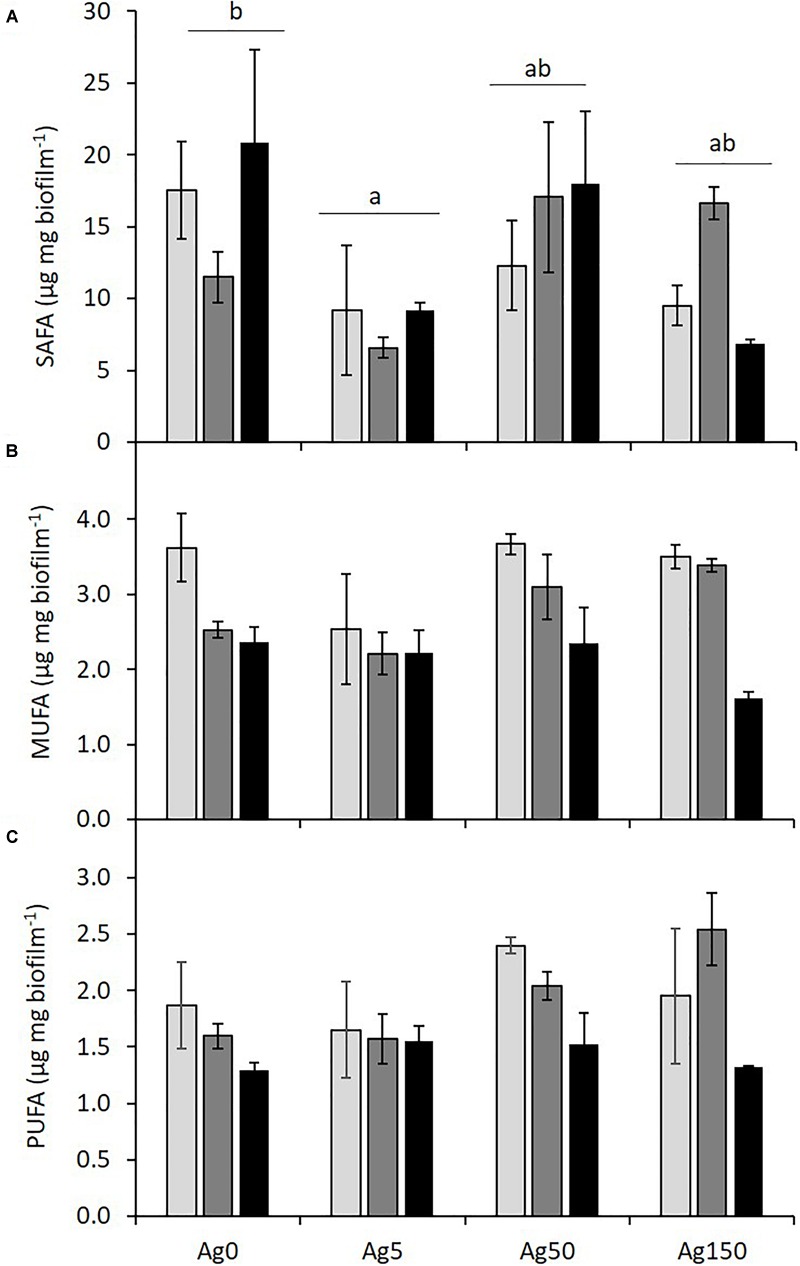
Biofilm contents (μg mg^-1^) of saturated (SAFA, **A**), monounsaturated (MUFA, **B**), and polyunsaturated (PUFA, **C**) fatty acids for the different P and Ag level tested. Significant differences due to Ag levels after *post hoc* Tukey tests are shown by different letters. No interactive effects were evidenced.

Fatty acids profiles (Table 2 and Figure 3) were slightly modified by both Ag and P exposures, but no interactive effects between both factors were evidenced. Culture medium P concentrations significantly reduced biofilm unsaturated fatty acid contents: MUFA concentrations were reduced by 36% (Figure 3B) while PUFA concentrations (Figure 3C) decreased by 28% between the P20 and the P500 treatments, respectively. The 20:5ω3 followed a similar trend, but differences were only marginally significant (*p* = 0.06). In contrast, low Ag contamination (5 μg L^-1^) led to a significant twofold decrease in biofilm SAFA content, while this reduction was no more significant for the 50 and the 150 μg Ag L^-1^ treatments. The same effect was observed on the total amount of fatty acids found in biofilm (Table 1).

**Table 2:** Results of the fatty acids analyses carried out on the biofilms at the end of the experiment.

Fatty acids (μg mg^-1^)	Ag0			Ag5			Ag50			Ag150		
	P20	P100	P500	P20	P100	P500	P20	P100	P500	P20	P100	P500
14:0	0.7 ± 0.0	0.6 ± 0.1	0.6 ± 0.1	0.5 ± 0.2	0.5 ± 0.1	0.6 ± 0.1	0.7 ± 0.0	0.7 ± 0.2	0.7 ± 0.3	0.7 ± 0.0	0.8 ± 0.1	0.6 ± 0.2
15:0		0.1 ± 0.0	0.1 ± 0.0	0.1 ± 0.0	0.1 ± 0.0	0.1 ± 0.0	0.1 ± 0.0	0.1 ± 0.0	0.1 ± 0.0	0.1 ± 0.0	0.1 ± 0.0	0.1 ± 0.0
16:0	8.6 ± 2.8	5.5 ± 1.3	9.6 ± 5.1	4.5 ± 2.9	3.2 ± 0.6	4.4 ± 0.5	6.1 ± 2.4	8.2 ± 4.1	8.3 ± 4.0	5.0 ± 1.0	7.9 ± 0.9	3.3 ± 0.3
17:0		0.1 ± 0.0	0.1 ± 0.0				0.1 ± 0.0			0.1 ± 0.0		
18:0	8.0 ± 3.1	5.0 ± 1.8	10.2 ± 6.0	4.0 ± 3.0	2.5 ± 0.5	3.9 ± 0.3	5.1 ± 2.9	7.9 ± 4.9	8.6 ± 4.3	3.5 ± 1.5	7.6 ± 1.1	2.8 ± 0.3
20:0	0.7 ± 0.0	0.1 ± 0.0	0.2 ± 0.1	0.1 ± 0.1	0.1 ± 0.0	0.1 ± 0.0	0.1 ± 0.0	0.1 ± 0.1	0.2 ± 0.1	0.1 ± 0.0	0.2 ± 0.0	0.1 ± 0.0
22:0	0.1 ± 0.0	0.1 ± 0.0	0.1 ± 0.0	0.1 ± 0.0		0.1 ± 0.0	0.1 ± 0.0	0.1 ± 0.0	0.1 ± 0.0	0.1 ± 0.0	0.1 ± 0.0	0.1 ± 0.0
24:0	0.1 ± 0.0	0.1 ± 0.0	0.1 ± 0.0	0.1 ± 0.0	0.1 ± 0.0	0.1 ± 0.0	0.1 ± 0.0	0.1 ± 0.0	0.1 ± 0.0	0.1 ± 0.0	0.1 ± 0.0	0.1 ± 0.0
**SAFA**	**17.5 ± 5.9**	**11.5 ± 3.1**	**20.9 ± 11.2**	**9.2 ± 6.4**	**6.6 ± 1.2**	**9.2 ± 0.9**	**12.3 ± 5.4**	**17.1 ± 9.1**	**18.0 ± 8.7**	**9.5 ± 2.4**	**16.6 ± 2.0**	**6.8 ± 0.6**
16:1ω9	0.1 ± 0.0	0.1 ± 0.0	0.1 ± 0.0	0.1 ± 0.0	0.1 ± 0.0	0.1 ± 0.0	0.2 ± 0.0	0.1 ± 0.1	0.2 ± 0.1	0.2 ± 0.0	0.2 ± 0.1	0.2 ± 0.0
16:1ω7	1.8 ± 0.4	1.2 ± 0.2	1.1 ± 0.3	1.3 ± 0.4	1.2 ± 0.3	1.3 ± 0.4	1.8 ± 0.3	1.4 ± 0.5	1.0 ± 0.3	1.5 ± 0.2	1.6 ± 0.1	0.7 ± 0.2
18:1ω9	1.3 ± 0.2	0.9 ± 0.0	0.9 ± 0.4	0.8 ± 0.4	0.6 ± 0.1	0.6 ± 0.1	1.3 ± 0.1	1.3 ± 0.3	0.9 ± 0.3	1.5 ± 0.1	1.2 ± 0.1	0.5 ± 0.0
18:1ω7	0.4 ± 0.2	0.3 ± 0.1	0.3 ± 0.0	0.3 ± 0.2	0.3 ± 0.1	0.2 ± 0.0	0.4 ± 0.0	0.3 ± 0.1	0.3 ± 0.1	0.3 ± 0.0	0.3 ± 0.0	0.2 ± 0.0
22:1ω9	0.1 ± 0.0											
**MUFA**	**3.6 ± 0.8**	**2.5 ± 0.2**	**2.4 ± 0.3**	**2.5 ± 1.0**	**2.2 ± 0.5**	**2.2 ± 0.5**	**3.7 ± 0.2**	**3.1 ± 0.8**	**2.4 ± 0.8**	**3.5 ± 0.3**	**3.4 ± 0.2**	**1.6 ± 0.2**
16:2ω4	0.2 ± 0.1	0.1 ± 0.0	0.1 ± 0.0	0.2 ± 0.1	0.1 ± 0.0	0.1 ± 0.0	0.2 ± 0.0			0.1 ± 0.1	0.1 ± 0.0	
16:4ω3	0.2 ± 0.0	0.1 ± 0.0	0.1 ± 0.0	0.1 ± 0.0	0.1 ± 0.1	0.1 ± 0.0	0.2 ± 0.0	0.1 ± 0.0	0.1 ± 0.0	0.1 ± 0.1	0.1 ± 0.0	0.1 ± 0.0
18:2ω6	0.5 ± 0.1	0.4 ± 0.0	0.3 ± 0.1	0.4 ± 0.2	0.3 ± 0.1	0.2 ± 0.0	0.5 ± 0.0	0.5 ± 0.1	0.3 ± 0.1	0.5 ± 0.2	0.5 ± 0.1	0.3 ± 0.0
18:3ω6	0.1 ± 0.0	0.1 ± 0.0		0.1 ± 0.0			0.1 ± 0.0	0.1 ± 0.0		0.1 ± 0.0	0.1 ± 0.0	
**18:3ω3**	**0.4 ± 0.2**	**0.4 ± 0.0**	**0.3 ± 0.0**	**0.3 ± 0.1**	**0.3 ± 0.1**	**0.4 ± 0.1**	**0.5 ± 0.0**	**0.6 ± 0.1**	**0.5 ± 0.1**	**0.5 ± 0.2**	**0.7 ± 0.2**	**0.6 ± 0.0**
20:4ω6	0.1 ± 0.0	0.1 ± 0.0	0.1 ± 0.0	0.1 ± 0.0	0.1 ± 0.0	0.1 ± 0.0	0.1 ± 0.0	0.1 ± 0.0	0.1 ± 0.0	0.1 ± 0.0	0.2 ± 0.1	0.1 ± 0.0
**20:5ω3**	**0.5 ± 0.3**	**0.4 ± 0.1**	**0.4 ± 0.1**	**0.6 ± 0.2**	**0.5 ± 0.1**	**0.5 ± 0.1**	**0.8 ± 0.1**	**0.7 ± 0.1**	**0.5 ± 0.2**	**0.6 ± 0.4**	**0.9 ± 0.3**	**0.4 ± 0.0**
22:6ω3		0.1 ± 0.0								0.1 ± 0.0		
**PUFA**	**1.8 ± 0.6**	**1.6 ± 0.2**	**1.3 ± 0.1**	**1.7 ± 0.6**	**1.6 ± 0.4**	**1.6 ± 0.2**	**2.4 ± 0.1**	**2.0 ± 0.2**	**1.5 ± 0.5**	**2.0 ± 1.0**	**2.5 ± 0.6**	**1.3 ± 0.0**
**Sum FA**	**23.0 ± 5.4**	**15.6 ± 2.7**	**24.5 ± 11.4**	**13.4 ± 8.0**	**10.4 ± 1.8**	**13.0 ± 1.6**	**18.3 ± 5.1**	**22.2 ± 9.5**	**21.9 ± 9.8**	**15.0 ± 2.6**	**22.6 ± 2.2**	**9.8 ± 0.6**

*Results are presented in μg of compound per mg of biofilm dry weight (±SE, *n* = 3). Data in bold indicate the most important classes of fatty acids.*

**FIGURE 4:**
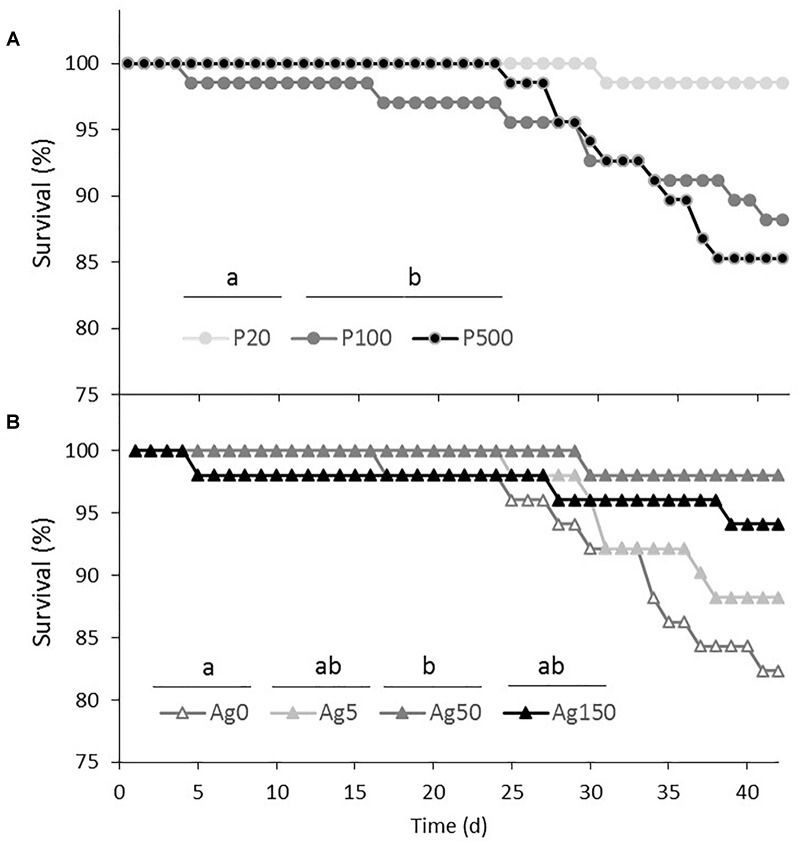
Survival curves of gammarids fed for 42 days with the different biofilms. Data represent cumulative survival of **(A)** all gammarids fed with biofilms coming from the P20, P100, and P500 treatments, whatever the Ag level, and **(B)** of all gammarids fed with biofilms coming from the Ag0, Ag5, Ag50, and Ag150 treatments, whatever the P level (see “Materials and Methods” section for justification). The different letters above the treatment names indicate significant differences in the survival curves after two by two curves comparisons, and Bonferroni corrections of the threshold *p*-values accounting for the multiple comparisons.

During the *G. fossarum* growth experiment, that lasted 42 days, the survival rate remained high (>80%) for all treatments. The survival was significantly lower for organisms fed with the biofilms coming from the P100 and the P500 treatments when compared to those fed with the biofilm grown in the P20 treatment (Figure 4A). Ag also had significant effects on gammarids survival, the lowest survival being observed for organisms fed with control (Ag0) biofilms, and the highest for organisms fed with biofilms coming from the Ag 50 treatment, those fed with biofilms coming from the Ag5 and the Ag150 treatments showing intermediate survival (Figure 4B).

**FIGURE 5:**
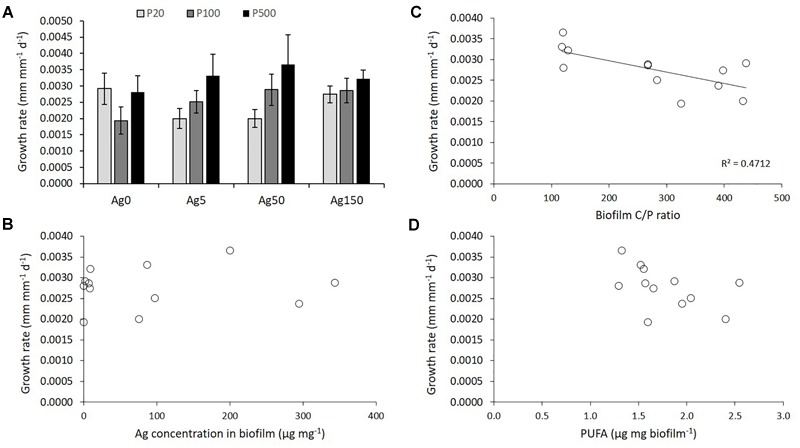
**(A)** Size growth rates of gammarids fed for 42 days on the different biofilms coming from the different P and Ag treatments. Relationships between Ag concentrations in biofilm **(B)**, biofilm P content **(C)** and biofilm PUFA content **(D)** and gammarids growth rates.

Size growth (calculated on the surviving organisms after the 42-days experiment) of organisms fed with the different biofilms was neither affected by the Ag nor by the P concentrations of biofilm exposure (Figure 5A), despite a marginally significant effect of P (*P* = 0.052, Table 1). When considering the regressions between organisms growth rates and the main descriptors of resource quality (PUFA and Ag concentrations, biofilm C:P ratios; Figure 5B–D), a significant negative relationship was only revealed for the effect of biofilm C:P ratios (*P* < 0.01) on *G. fossarum* growth, whereas no significant relationship was found for biofilm Ag and PUFA concentrations.

## Discussion

While the single and interactive impacts of nutrient concentrations and metallic contaminations have long been investigated on biofilm community structures (Guasch et al., 2004; Tlili et al., 2010; Serra et al., 2010; Murdock and Wetzel, 2012; Murdock et al., 2013; Leflaive et al., 2015), far less is known on the effects these multiple stressors might play on the quality of the biomass produced, and, in turn, on their consequences for biofilm consumers. In this study, we showed that both phosphorus and silver significantly change diverse parameters of biofilms quality for an invertebrate consumer, without strong interactive effects between stressors. In particular, we partially verified Hypothesis 1, P but not Ag significantly reducing biofilm PUFA content. The P-level also markedly increased biofilm elemental quality, validating Hypothesis 2. In contrast, Hypothesis 3, dealing with the negative effects of Ag contaminated resources ingestion on consumers must be rejected.

### Conditions of Biofilm Exposure to Ag

Despite significant initial differences in Ag concentrations in water between the four Ag treatments, Ag concentrations measured at t0 were systematically lower than nominal concentrations (differences with expected concentrations ranging from 1% in the Ag50 treatment to 37% and 48% in the Ag150 and the Ag5 treatments, respectively). This observation suggests that a part of the metal might have been very quickly adsorbed to the biofilm and/or to the microcosms walls at the microcosm filling. The lack of interaction with P level as well as the high recovery of dissolved silver in the culture medium (e.g., >80% for the 150 μg Ag L^-1^, P500 treatment) suggests that a fast complexation and a precipitation in presence of phosphates, if it occurred, might have been reduced.

After 1 week of biofilm exposure to silver (*t*_final_), silver concentrations were largely reduced when compared to what was initially introduced. These reductions could be partly attributed to silver uptake by biofilm microorganisms (algae, bacteria, fungi…), but also to the great potential of growing algal cells and biofilms to adsorb ionic metals on their surfaces. Indeed, adsorption processes are generally more common in algae than active uptake into the cells (Ratte, 1999). Adsorption has already been shown to increase with the presence of extracellular polymers (EPS), as already observed in previous studies (e.g., González et al., 2015, 2016), such substances presenting numerous potential binding sites (van Hullebusch et al., 2003). Since nutrient depletions are well known to stimulate the production of EPS by phototrophic biofilms (Lyon and Ziegler, 2009), an increase in Ag adsorption was expected in the P20 treatment when compared to the P500 treatment. However, in our study, lower P levels did not change significantly silver concentrations remaining in water. Similarly, even if we were unable to test for the interactive effects of P and Ag on silver concentration in biofilms (absence of replication for these chemical analyses), higher P concentrations in water were expected to lead to reduced Ag concentrations in biofilms. Again, this general trend was not clearly visible, and complementary studies will be required to understand the mechanisms explaining the changes in silver concentrations, both in the water column and in the biofilm.

### Effects of P and Ag on Biofilm Elemental Composition

Elemental compositions of resources are generally considered as good proxies of resources quality for consumers (Sterner and Elser, 2002). Indeed, life history traits of several species have been shown to be controlled by the imbalance between their elemental requirements and what they can effectively find in their resources (e.g., Elser et al., 2001; Frost and Elser, 2002). Since C proportions in resources is rarely limiting for consumers, resources presenting low C:N and/or low C:P ratios are generally considered as potentially high quality resources for their consumers (Sterner and Elser, 2002). Our study revealed that both biofilm C:P and N:P ratios were significantly reduced by the P concentration of the culture medium. This effect might be simply explained by the fact that algae have the potential to immobilize nutrients in excess in their biomass, this process being generally called luxury consumption (Droop, 1974). This effect occurs concomitantly with a reduction of the C-rich EPS production (Lyon and Ziegler, 2009), these compounds generally largely contributing to increasing algal communities C:nutrient ratios (Pannard et al., 2016). In addition, bacterial communities have the potential to quickly change, selecting for species able to use optimally nutrients available and adjusting the stoichiometry of the whole community to that of their resources (Danger et al., 2008). Unexpectedly, silver contamination also led to significant reductions of biofilm C:N and C:P ratios, even if these reductions remained low when compared to those observed along the P gradient. Data on the effects of contaminants on resources elemental compositions remain extremely scarce in the literature. Some previous studies showed that biofilms tend to increase their production of C-rich EPS as a response to contaminant exposure (García-Meza et al., 2005; Serra and Guasch, 2009; González et al., 2016). This physiological response, aimed at increasing the metal-binding sites of the biofilm, would thus be expected to increase biofilm C:nutrient ratios (Pannard et al., 2016). In our study, the opposite effects were observed. This result might thus alternatively be explained by the large Ag-induced shifts in prokaryotic and microeukayotic community structures observed in our study (see Leflaive et al., 2015). One could expect that different species with different luxury consumption capabilities might be selected by Ag contamination. Another potential, non-exclusive, explanation could be that organisms stressed by the Ag contamination tend to increase their antitoxic defenses, these defenses generally relying on N-rich enzymes and leading to apparent lower biofilm C:N ratios.

### Effects of P and Ag on Biofilm Fatty Acid Profiles

Some fatty acids, and especially long-chain PUFAs have been described as pertinent indicators of resources quality. Indeed, most metazoans are unable to (or, at least, not in sufficient amounts) synthesize these essential compounds that must be found in their diet. These compounds are notably involved in the synthesis of key hormones and represent important molecules in cell membranes, controlling in particular membrane fluidity (Arts et al., 2009). In algal communities, diatoms are known to produce high amounts of long chain PUFAs (20:5ω3, Dunstan et al., 1994) in comparison with green algae and cyanobacteria that mainly produce shorter chain PUFAs (Masclaux et al., 2009) or are unable to synthesize highly unsaturated fatty-acids (Muller-Navarra et al., 2004), respectively. In the present study, both Ag contamination and P concentrations greatly altered algal communities, reducing diatoms proportion and increasing green algae proportions (see Leflaive et al., 2015, Figure 1). Both P and Ag stressors led to similar effects, without acting interactively. Thus, reductions in biofilm PUFA and 20:5ω3 content were expected for both stressors. In the present study, only the P increase led to significant reductions in these compounds. Surprisingly, we did not observe any significant effect of silver on biofilm PUFA and 20:5ω3 contents. In contrast, silver contamination significantly modified biofilm SAFA and the total amount of fatty acids, these parameters reaching their minimal values for an exposure to 5 μg Ag L^-1^. The effect of P level on biofilm fatty acid profiles can certainly be explained by the replacement of 20:5ω3-rich diatoms by green algae, as already observed in diverse studies dealing with natural communities (e.g., Muller-Navarra et al., 2004; Bec et al., 2010). The absence of silver effect on biofilm PUFA and 20:5ω3 contents, despite the replacement of diatoms by green algae, could be explained either by the replacement of some diatom species by other diatom species containing more PUFAs or by an increase of diatoms’ PUFA content as a response to Ag contamination. However, Ag and P stressors led to similar changes in biofilm community compositions (Leflaive et al., 2015), both communities showing similar reductions in the abundance of the dominant diatom species (*Achnanthidium minutissima*, Kützing and *Cymbella excisa*, Kützing). The second explanation thus appears as the most probable. Even if data remain scarce in the literature, such an effect of fatty acid synthesis deregulation was already found in the macroalgae *Fucus* sp. exposed to copper (Smith et al., 1985). Similarly, some studies showed that fatty acid profiles of unicellular organisms (Green algae: McLarnon-Riches et al., 1998; Diatom: Jones et al., 1987; Euglenophyceae: Rocchetta et al., 2006) were susceptible to change after an exposure to metals. Yet, there is still no consensus on the toxic metals effects on algae fatty acids profiles, some studies showing reductions in long chain PUFAs due to reductions in their abundance or synthesis caused by oxidative stress (Rocchetta et al., 2006), while other studies showed increases in algal PUFAs after alterations of enzymatic activities (McLarnon-Riches et al., 1998). The precise understanding of silver impacts on biofilm fatty acid contents would greatly benefit from specific investigations of fatty acid synthesis on algal communities.

### Effects of P and Ag on Biofilm Quality for an Invertebrate Consumer

While biofilm Ag, nutrients, and PUFA concentrations remain only potential quality parameters for biofilm consumers, measuring the impacts of biofilms consumption on metazoans is the only way for evaluating the effective quality of biofilms, and anticipate the indirect effects of the multiple stressors selected (Ag and P) on higher trophic levels. Consumers’ growth measurements have been shown as a powerful mean for evaluating resources quality. In particular, effects of resources stoichiometry and/or highly unsaturated fatty acids on consumers’ growth have already been successfully tested on planktonic and terrestrial herbivores (e.g., Elser et al., 2001; Schade et al., 2003; Masclaux et al., 2009). More recently, such effects have been revealed in the crustacean species, *Gammarus fossarum*, both for the effect of resources P (Danger et al., 2013) and highly unsaturated fatty acid contents (Crenier et al., 2017). In the present study, biofilm C:P ratios were significantly related to *G. fossarum* growth, growth being reduced when organisms were fed with the highest C:P ratio resources. Organisms P content being directly related to organisms’ nucleic acid production and cell proliferation (Elser et al., 2003), eating low C:P resources can help organisms to overcome P limitations of their growth. Changes in microbial biomass PUFA and 20:5ω3 contents induced by biofilm exposure to high P concentrations, while significant, were too small for detecting any significant effect on consumers’ growth. Finally, contrary to expectations, accumulation of Ag in biofilm biomass had strictly no influence on consumers’ growth. Yet, some studies showed that toxicity of Ag on zooplankton species could be higher via trophic transfer than by direct uptake from water (Hook and Fisher, 2001; Bielmyer et al., 2006). In contrast, other studies suggested that Ag toxicity was very variable depending on water chemistry, and that free ionic silver was the most toxic form to invertebrates (Ratte, 1999). In our study, elemental quality of resources seemed thus to be more important than biofilm silver concentration for *G. fossarum*. However, it must be noted that responses could have been different if other life history traits had been considered. For example, PUFA concentrations have been regularly shown to control organisms’ reproduction (e.g., Masclaux et al., 2009), and multiple stressor effects might also impact organisms reproduction.

Finally, it must be noted that in contrast to the observed stimulation of *G. fossarum* growth, our results also showed a low but significant negative effect of P on organisms’ survival. Such an effect has already been observed in another study (Arce-Funck et al., 2018). It was proposed that higher growth rates generated by higher resource quality increases molting frequency. Yet, molting is by far the most sensitive stage of molting organisms’ development, especially when exposed to contaminants (McCahon and Pascoe, 1988). Stimulation of organisms’ growth thus generally co-occur with an increase in organisms’ mortality. In contrast, the highest survival found for Ag-fed gammarids is more difficult to explain. One could imagine a stimulation of immune defenses of gammarids or a reduction of potentially pathogenic bacteria growing in biofilms. Such effects yet remains to be tested.

## Conclusion

To conclude, our results showed that both P and Ag impacted several biofilm quality parameters, but never interactively. The use of *G. fossarum* growth experiment permitted to verify the consequences of microbial resources potential quality. In this study, resources C:P was the most important parameter. Similar studies would be required to understand in more details the indirect effects multiple stressors might play on microorganisms consumers and, in turn, on ecosystem functioning.

## Author Contributions

MD, AB, VF, LT-H, and JL designed the experiments. CC, JL, JF, and KS-T carried out the experiments. CC, AG, FP, and JF carried out chemical analyses. All authors contributed to the writing of the manuscript.

## Conflict of Interest Statement

The authors declare that the research was conducted in the absence of any commercial or financial relationships that could be construed as a potential conflict of interest.
